# Concurrent minimal change nephrotic syndrome and type 1 diabetes mellitus in an adult Japanese woman: a case report

**DOI:** 10.1186/s12882-020-02071-6

**Published:** 2020-09-23

**Authors:** Ryuzoh Nishizono, Hiroki Kogou, Yuri Ishizaki, Akihiro Minakawa, Masao Kikuchi, Hiroko Inagaki, Yuji Sato, Shouichi Fujimoto

**Affiliations:** 1 Department of Nephrology, Faculty of Medicine, University of Miyazaki, Miyazaki, Japan; 2 Dialysis Division, University of Miyazaki Hospital, Miyazaki, Japan; 3 Department of Hemovascular Medicine and Artificial Organs, Faculty of Medicine, University of Miyazaki, Miyazaki, Japan

**Keywords:** Type 1 diabetes mellitus, Steroid-sensitive nephrotic syndrome, Minimal change nephrotic syndrome, Genetic factors

## Abstract

**Background:**

Concurrent type 1 diabetes mellitus (T1DM) and idiopathic nephrotic syndrome is rare, and most previously reported cases were in children. We report the case of an adult woman who developed T1DM and minimal change nephrotic syndrome (MCNS) nearly simultaneously.

**Case presentation:**

A 24-year-old woman had first presented to another hospital with nausea, vomiting, and fatigue. She was diagnosed with diabetic ketoacidosis and T1DM on the basis of her hyperglycemia, ketoacidosis, and positive anti-glutamic acid decarboxylase antibody test result. Rapid infusion of normal saline and insulin administration alleviated hyperglycemia and ketoacidosis. Two weeks after admission, however, she developed nephrotic syndrome (NS) with rapidly decreasing urine volume. She was referred to our hospital with a diagnosis of acute kidney injury. Although she temporarily required dialysis and high doses of insulin, within 1 month NS and acute kidney injury had been alleviated by oral prednisolone and low-density lipoprotein apheresis. Renal biopsy showed minor glomerular abnormalities without diabetic nephropathy, so we diagnosed her with MCNS. Seven weeks after the discharge, NS relapsed, and cyclosporine was added to prednisolone. However, NS relapsed twice within the next 4 months, so we started her on rituximab. At 6 months after initiating rituximab therapy, she remained in complete remission.

Her mother also had T1DM but not MCNS. The patient had HLA-DRB1*09:01/09:01, DQB1*03:03/03:03, and her mother had HLA-DRB1*04:05/09:01, DQB1*03:03/04:01.

**Conclusions:**

Concurrent T1DM and MCNS is rare and their coexistence might be coincidental. Alternatively, they might have been caused by an underlying, unidentified genetic predisposition. Previous reports and our patient’s findings suggest that specific HLA alleles and haplotypes or a Th1/Th2 imbalance might be associated with T1DM and MCNS that occurred nearly simultaneously.

## Background

Type 1 diabetes mellitus (T1DM) and idiopathic nephrotic syndrome are more common in children than in adults. The incidence of T1DM in Japanese children < 14 years of age is 1.4–2.25/100,000 [1], which is much lower than that in other countries, especially Finland (45.0–64.2/100,000 children < 15 years of age) [2]. Conversely, the estimated incidence of pediatric idiopathic nephrotic syndrome in Japan is 6.49 cases/100,000 [3], which is slightly higher than the average incidence in previous studies worldwide (4.7/100,000) [4]. Most cases of pediatric idiopathic nephrotic syndrome manifest as minimal change nephrotic syndrome (MCNS) in Japan, which is similar to that in other countries.

Some case reports described children who had developed idiopathic nephrotic syndrome soon after being diagnosed with T1DM, whereas others, conversely, had developed T1DM soon after being diagnosed with idiopathic nephrotic syndrome [5–11]. Most patients with MCNS had neither diabetic nephropathy nor retinopathy. We report a rare case in which an adult woman developed T1DM and MCNS nearly simultaneously. We then discuss the patient’s genetic background.

## Case presentation

A 24-year-old Japanese woman was admitted to another hospital with nausea, vomiting, and general fatigue. She had a 2-month history of strongly feeling thirsty, and she had lost 5 kg during that period. She had no history of infectious diseases such as bronchitis before feeling thirsty. Her only medical history was seasonal allergic rhinitis, but her family history included T1DM. Her mother was diagnosed with T1DM at 29 years of age. Blood tests in the present patient showed a high blood glucose level (340 mg/dL), ketoacidosis (pH 7.21; bicarbonate [HCO_3_], 8 mmol/L; anion gap, 21.3 mmol/L), and hemoglobin A1c (12.7%). The anti-glutamic acid decarboxylase antibody test result was 708 U/mL, and she was diagnosed with diabetic ketoacidosis and T1DM.

Her symptoms, hyperglycemia, and ketoacidosis were alleviated by rapid normal saline infusion and insulin administration. Shortly after the first insulin lispro injection, however, she exhibited an itchy eruption, and the insulin lispro was changed to insulin aspart. Insulin glargine was also added. Two weeks after admission, the patient developed nephrotic syndrome, and her urine volume decreased rapidly. She was referred to our hospital with a diagnosis of acute kidney injury.

On admission, she was found to have gained 4 kg in weight within the past 2 weeks due to oliguria. Physical examination showed lower-leg pitting edema. Her blood pressure was 106/66 mmHg, and urinalysis and blood test results revealed findings that were consistent with nephrotic syndrome and renal dysfunction (Table 1). Her serum immunoglobulin E (IgE) level was very high (4882 IU/mL), and there was no eosinophilia.

**Table 1:** Patient’s laboratory data at admission

**<Urinalysis>**	
**Urinary protein (g/day)**	**10**
**Urinary C-peptide (**μg**/day)**	**17 (24–97)**
**<Blood test>**	
**Total protein (g/dL)**	**4.83**
**Serum albumin (g/dL)**	**1.28**
**Blood urea nitrogen (mg/dL)**	**54.0**
**Serum creatinine (mg/dL)**	**4.04**
**Total cholesterol (mg/dL)**	**434**
**Plasma glucose (mg/dL)**	**85**
**Hemoglobin A1c (%)**	**12.7**
**Serum C-peptide (ng/mL)**	**2.0 (1.2–2.0)**
**Serum IgG (mg/dL)**	**479 (861–1747)**
**Serum IgE (IU/mL)**	**4882 (3.7–311.6)**
**Free triiodothyronine (FT3) (pg/mL)**	**1.5 (1.88–3.18)**
**Free thyroxine (FT4) (ng/dL)**	**0.78 (0.7–1.48)**
**Thyroid stimulating hormone (μIU/L)**	**1.01 (0.35–4.94)**
**Thyroglobulin antibody**	**negative**
**Thyroid peroxidase antibody**	**negative**
**Ant-GAD antibody (U/mL)**	**708 (< 5.0)**
**Anti-IA-2 antibody (U/mL)**	**6.3 (< 0.6)**
**Anti-insulin antibody**	**negative**
**Anti-ZnT8 antibody**	**negative**
**Anti-islet cell antibody**	**negative**

() normal range.

*Ig* Immunoglobulin, *GAD* Glutamic acid decarboxylase, *IA-2* Insulinoma-associated antigen-2, *ZnT8* Zinc transporter 8

**Fig. 1:**
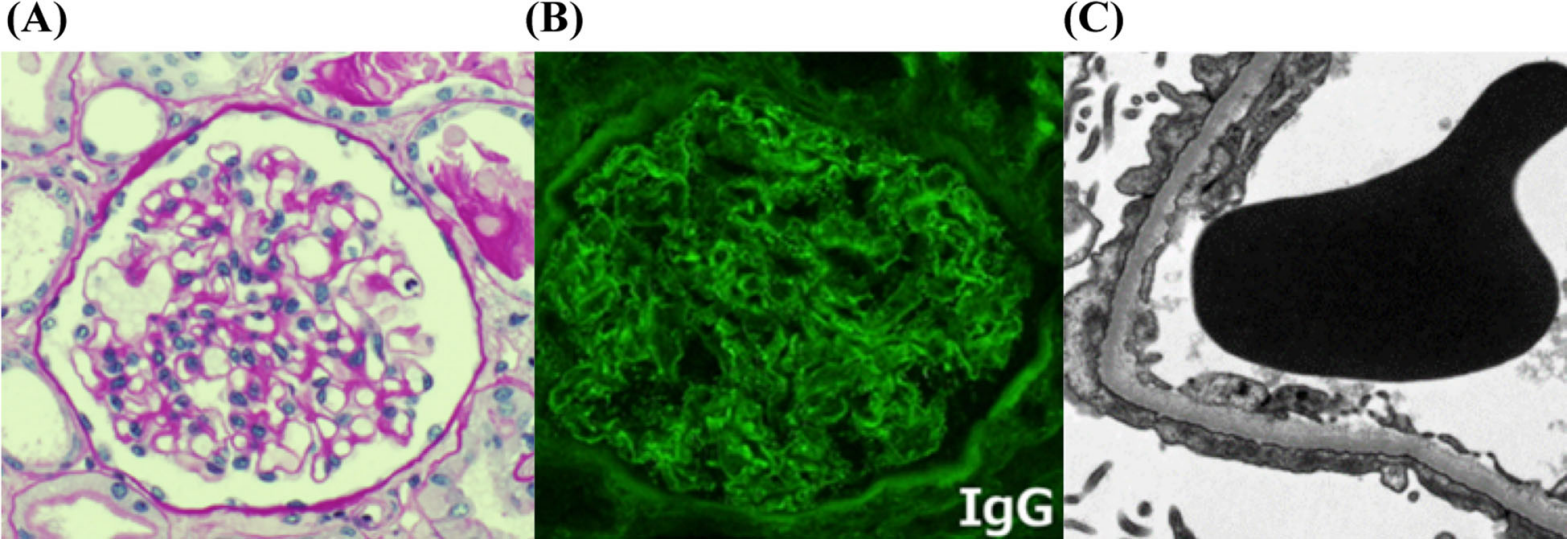
Micrographs of renal biopsy findings. **a** Light micrograph of a glomerulus shows no evidence of diabetic nephropathy (periodic acid-Schiff stain). **b** Glomerulus shows positive immunofluorescence staining for immunoglobulin G (IgG) along a capillary wall. **c** Electron micrograph shows effacement of the podocyte foot process and a glomerular capillary membrane of normal thickness without evidence of capillary immune complex deposits. (A × 400; B × 400; C × 5000)

Insulin treatment for T1DM was continued, and hemodialysis was initiated on day 2 and continued three times per week because of acute kidney injury. A renal biopsy was performed on day 15 after admission. Among the 46 glomeruli that were obtained in the biopsy, one glomerulus showed global sclerosis, and the others showed some mesangial matrix expansion but without hypercellularity or extracapillary or endocapillary proliferation. The pathological diagnosis was minor glomerular abnormalities including immunofluorescence and electron micrographic studies (Fig. 1), and we diagnosed her with MCNS.

**Fig. 2:**
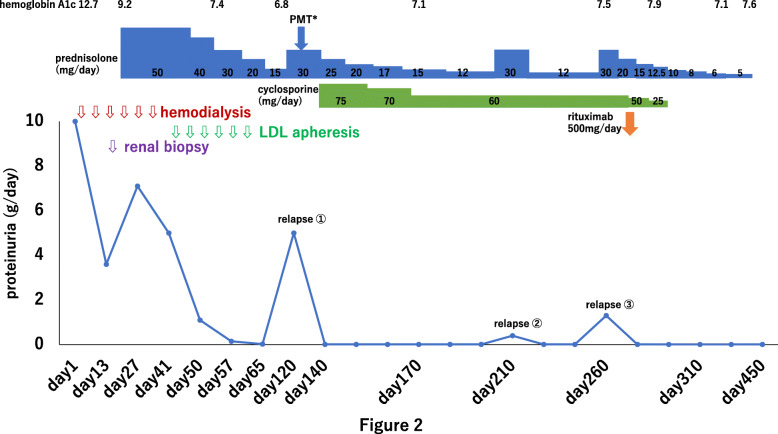
Clinical course. *PMT, pulse methylprednisolone therapy: 500 mg of methylprednisolone intravenously for 3 days

The patient’s clinical course is shown in Fig. 2. Treatment with oral prednisolone (PSL) at a dose of 0.8 mg/kg (50 mg/day) was started on day 22. Her urinary volume increased within 2 weeks, allowing discontinuation of hemodialysis on day 38, although her urinary protein remained at 5 g/day. Because nephrotic syndrome is not a result of MCNS but, rather, it is caused by focal segmental glomerulosclerosis (FSGS), we started low-density lipoprotein apheresis (LDL-A) on day 42. LDL-A was performed twice a week for 3 weeks. Her urinary protein decreased, and the urine volume gradually increased. On day 55, she was in complete remission. PSL was tapered by 10 mg every 2 weeks, and she was discharged from the hospital at a reduced PSL dose of 30 mg/day on day 71.

Seven weeks later, the patient had a relapse of nephrotic syndrome, and cyclosporine 1.5 mg/kg (75 mg/day) was added to PSL. However, NS relapsed twice within the next 4 months, so we started her on a single dose of rituximab (375 mg/m^2^) after explaining the prognosis of frequently relapsing nephrotic syndrome. Serum CD19/CD20 levels decreased to 0.1/0.0% after 1 month, and we discontinued cyclosporine. PSL was tapered from 30 mg/day to 5 mg/day over 6 months, and hemoglobin A1c remained at 7.1–7.9% with insulin treatment. At 6 months after initiating rituximab, she remained in complete remission without any reported side effects.

To investigate the genetic background of the co-occurrence of both diseases, we performed an HLA testing on the patient and her mother. The patient had HLA-DRB1*09:01/09:01, DQB1*03:03/03:03, and her mother had HLA-DRB1*04:05/09:01, DQB1*03:03/04:01.

## Discussion and conclusions

We report the rare case of a young woman who developed T1DM and MCNS nearly simultaneously. We later found that her mother also had T1DM. The patient had no diabetic retinopathy or nephropathy, but her hemoglobin A1c was 12.7%. A previous report noted that the glomerular basement membrane was partially thickened in a patient 6 months after being diagnosed with diabetes mellitus [12, 13], but it was not thickened in our patient. A possible explanation is that she had developed subclinical acute-onset T1DM a few months before admission, which had manifested as a strong feeling of thirst during the 2 months before her admission. She had also lost weight (5 kg).

Although renal biopsy showed minor glomerular abnormalities, it might have been FSGS because she required LDL-A as the initial treatment and her nephrotic syndrome relapsed several times. She had no diabetic nephropathy, but a trace of linear IgG deposition in the capillary walls was revealed by immunofluorescence staining. We could not determine whether the deposition was an immunological reaction or a non-specific change that is occasionally seen in MCNS/FSGS patients.

In the present case, we could control her hemoglobin A1c level, which was 7.1–7.9% after rituximab treatment despite T1DM and chronic steroid use. Additionally, the insulin dose did not need to be increased. Is immune intervention beneficial in T1DM patients? Some previous studies showed beneficial effects of immunosuppressants including rituximab in T1DM [14–18], but longer-term follow-up revealed a decline in the effect over time [19–23]. It is uncertain whether good glycemic control in the present case resulted from rituximab, but rituximab had a steroid-sparing effect and potentially contributed to preventing hyperglycemia.

The association between T1DM and idiopathic nephrotic syndrome has been known for more than 6 decades, but there are few reports of patients who had co-occurrence of these conditions (Table 2). Although the nature of this association remains unclear, several hypotheses have been offered. A few reports described that HLA loci, especially HLA-DR/DQ, might be associated with a genetic predisposition for the development of both diseases (Table 3). However, we must take into consideration that the genetic background of T1DM and nephrotic syndrome patients in Japan is different from that of patients from other countries. In Caucasians, nephrotic syndrome has been reported to be associated with HLA DR7, and the presence of the HLA-DRB1*03:01-DQB1*02:01 haplotype poses a high risk for developing T1DM. However, those HLA types are extremely rare in a general Japanese population [28].

**Table 2:** Summary of previous reports of patients with nephrotic syndrome with type 1 diabetes mellitus that developed within 1 year

References	Age at onset of T1DM (years)	Age at onset of NS (years)	Pathological diagnosis	Treatment	Outcome
Robinson [12]	8	8	Not done	Steroid, Diuretics	Resolved completely
Urizar [11]	4	4 (1 week after DM)	Normal	Insulin	Resolved completely
	8	8	Minimal focal glomerulitis	Steroid	Resolved completely
	3.3	4.3	Normal	Steroid	Resolved completely
	5	5	Minimal focal glomerulitis	Steroid	Recurrence
Robbinson [10]	3	3 (2 months after DM)	ICGN	Steroid	Resolved completely
Dornan [9]	20	20 (2 weeks after DM)	MCD	Diuretics	Resolved
	13	13 (1 week after DM)	Not done		Resolved spontaneously
Rego Filho [7]	3.9	3.9	Not done	Steroid, CPM	Resolved
Nakahara [23]	8	8	MCD	Steroid, CPM	?
Agras [24]	3	3 (10 months after DM)	Not done	Steroid, CPM	Resolved
Jameela [25]	2.75	2.75	Diffuse expansion of mesangial matrix	Steroid, CPM	Resolved
	1.5	1.5	Diffuse expansion of mesangial matrix	Steroid, CPM	Resolved
Otukesh [6]	13 days	13 days	MGN	?	?

*T1DM* Type 1 diabetes mellitus, *NS* Nephrotic syndrome, *CPM* Cyclophosphamide, *MGN* Membranous glomerulonephritis, *MCD* Minimal-change disease, *ICGN* Immune-complex glomerulonephritis

**Table 3:** HLA class II antigens and DNA typing from previous reports and the present patient, and all these patients developed nephrotic syndrome and type 1 diabetes mellitus concurrently

Case	HLA class II
Rego Filho [7]	DR 4, DR 8, DR 53
Peces [26]	DR 4, DR 7
Kagiyama [27]	DR 2, DR 9
Agras [24]	DR 4, DR 11, DR 52, DR 53, DQ 7, DQ 8
Present case	DR 9 (DRB 1*09:01), DQ 9 (DQB 1*03:03)

*HLA* Human leukocyte antigen

In the Japanese population, HLA-DRB1*04:05, HLA-DRB1*09:01, HLA-DQB1*04:01, and HLA-DQB1*03:03 alleles are associated with susceptibility to T1DM. Additionally, HLA-DRB1*04:05-DQB1*04:01, HLA-DRB1*08:02-DQB1*03:02, and HLA-DRB1*09:01-DQB1*03:03 haplotypes are associated with susceptibility to T1DM [29]. Recently, it has been reported that the HLA-DRB1*08:02, HLA-DQB1*03:02 alleles, and the HLA-DRB1*08:02-DQB1*03:02 haplotype is highly associated with steroid-sensitive nephrotic syndrome in Japanese children [30]. Although we had speculated that our patient would have HLA-DRB1*08:02-DQB1*03:02, she had HLA-DRB1*09:01/09:01 and DQB1*03:03/03:03. Her mother had HLA-DRB1*04:05/09:01 and DQB1*04:01/03:03. Both the patient and her mother had specific HLA haplotypes that are highly associated with T1DM, but only the daughter developed MCNS. HLA-DRB1*09:01-DQB1*03:03-DPB1*02:01 and HLA-A*02:06-C*08:01-B*40:06-DRB1*09:01-DQB1*03:03 haplotypes have been reported to be associated with childhood steroid-sensitive nephrotic syndrome in Japan, so our patient might have either one of the two haplotypes [30].

Another hypothesis that explains concurrent T1DM and MCNS is Th1/Th2 imbalance [31–35]. Both diseases have a strong association with T-cell abnormalities, although the mechanisms are different. MCNS is known to be associated with allergic disease [36], and it has been reported to be associated with prevalent type 2 helper T-cell (Th2) responses. The association of T1DM with autoimmune disorders is also well known [37], and its association with prevalent type 1 helper T-cell (Th1) responses has been reported. However, neither Th1 predominance in T1DM nor Th2 predominance in MCNS has not been definitively identified [38, 39].

Previous case reports described that insulin treatment influenced T-cell differentiation and promoted a shift toward a Th2-type response [40–42]. These previous reports strongly suggested that insulin could function as a glucose-regulatory hormone and as a T-cell hormone, which enhances the Th2 response and consequently increases the in vitro production of Th2 profile cytokines such as interleukin (IL)-4 and IL-10 in patients who are at high risk of DM and T1DM.

In the present case, the patient developed T1DM first, followed by MCNS 2 weeks later. We speculate that these diseases were manifest via two different mechanisms. First, insulin lispro (or aspart) administration might cause a drastic shift in the Th1 response to a Th2 response. Second, insulin lispro may have triggered an allergic reaction causing MCNS because she developed an itchy eruption shortly after the first insulin lispro injection and had a high level of serum IgE. No previous reports, however, have demonstrated the simultaneous onset of T1DM and MCNS, and due to a Th1/Th2 imbalance, and we did not measure the Th1/Th2 ratios and serum cytokine levels that were secreted by Th1 and Th2.

In summary, we report the rare case of an adult woman with acute kidney injury that resulted from MCNS, which had developed soon after she was diagnosed with diabetic ketoacidosis that was caused by T1DM. The mechanism by which T1DM and MCNS occurred concurrently remains unclear, although some previous reports and our case indicate that genetic factors (e.g., specific HLA alleles and haplotypes) or a Th1/Th2 imbalance might be associated with the onset of these two diseases.

## Supplementary information

**Additional file 1.**

## Data Availability

All data supporting the case are included in the manuscript.

## Abbreviations

T1DM: Type 1 diabetes mellitus
NS: Nephrotic syndrome
MCNS: Minimal change nephrotic syndrome
Ig: Immunoglobulin
GAD: Glutamic acid decarboxylase
IA-2: Insulinoma-associated antigen-2
ZnT8: Zinc transporter 8
CPM: Cyclophosphamide
LDL-A: Low-density lipoprotein apheresis
MGN: Membranous glomerulonephritis
MCD: Minimal-change disease
FSGS: Focal segmental glomerulosclerosis
ICGN: Immune-complex glomerulonephritis
HLA: Human leukocyte antigen

## References

[1] Onda Y, Sugihara S, Ogata T, Yokoya S, Yokoyama T, Tajima N (2017). Type 1 diabetes (T1D) study group. Incidence and prevalence of childhood-onset type 1 diabetes in Japan: the T1D study. Diabet Med.

[2] Songini M, Mannu C, Targhetta C, Bruno G (2017). Type 1 diabetes in Sardinia: facts and hypotheses in the context of worldwide epidemiological data. Acta Diabetol.

[3] Kikunaga K, Ishikura K, Terano C, Sato M, Komaki F, Hamasaki Y (2017). Japanese pediatric survey holding information of NEphrotic syndrome study of the Japanese study Group of Renal Disease in children (JP-SHINE study). High incidence of idiopathic nephrotic syndrome in east Asian children: a nationwide survey in Japan. Clin Exp Nephrol.

[4] Chanchlani R, Parekh RS (2016). Ethnic differences in childhood nephrotic syndrome. Front Pediatr.

[5] Bawahab NS, Safdar OY, Nagadi SA, Saeedi AT, Mohammed Hussain RW (2019). Nephrotic syndrome co-existing with type 1 diabetes in a 12-year-old boy: Case report and literature review. SAGE Open Med Case Rep.

[6] Otukesh H, Torabi A, Hoseini R, Rahimabad PK, Mehrazma M (2016). Co-existance of type 1 diabetes mellitus and nephrotic syndrome with membranous glomerulonephritis in a 6-year old boy: report of a case. Int J Children Adolesc.

[7] Rego Filho EA, Mello SF, Omuro AM, Loli JO (2003). Simultaneous onset of steroid-sensitive nephrotic syndrome and type 1 diabetes. J Pediatr.

[8] Goldman M, Hébert D, Geary DF (2002). Management of steroid-sensitive nephrotic syndrome in children with type 1 diabetes. Pediatr Nephrol.

[9] Dornan TL, Jenkins S, Cotton RE, Tattersall RB, Burden RP (1988). The nephrotic syndrome at presentation of insulin-dependent diabetes mellitus; cause or coincidence?. Diabet Med.

[10] Robinson LA, Howel DN, Wigfall DR, Foreman JW (1997). Appearance of immune complex glomerulonephritis following the onset of type 1 diabetes mellitus in a child. Am J Kidney Dis.

[11] Urizar RE, Schwartz A, Top F, Vernier RL (1969). The nephrotic syndrome in children with diabetes mellitus of recent onset. N Engl J Med.

[12] Robinson GC, McConnell D (1961). Simultaneous onset of diabetes mellitus and nephrotic syndrome. Can M A J.

[13] Goetz FC, Hartmann JF, Lazarow A (1960). Electron microscopy of the human glomerulus in early diabetes. J Clin Invest.

[14] Pescovitz MD, Greenbaum CJ, Krause-Steinrauf H, Becker DJ, Gitelman SE, Goland R (2009). Rituximab, B-lymphocyte depletion, and preservation of beta-cell function. N Engl J Med.

[15] Kurozumi A, Okada Y, Arao T, Miyazaki Y, Yoshikawa M, Torimoto K, et al. Pancreas-protective effect of rituximab for acute-onset type 1 diabetes in the honeymoon period: a case report. Endocrinol Diabetes Metab Case Rep. 2016;160020. 10.1530/EDM-16-0020. 10.1530/EDM-16-0020 PMC4870497 27252867

[16] Herold KC, Hagopian W, Auger JA (2002). Anti-CD3 monoclonal antibody in new-onset type 1 diabetes mellitus. N Engl J Med.

[17] Keymeulen B, Vandemeulebroucke E, Ziegler AG (2005). Insulin needs after CD3-antibody therapy in new-onset type 1 diabetes. N Engl J Med.

[18] Orban T, Bundy B, Becker DJ (2011). Type 1 diabetes TrialNet Abatacept study group co-stimulation modulation with abatacept in patients with recent-onset type 1 diabetes: a randomised, double-blind, placebo-controlled trial. Lancet.

[19] Pescovitz MD, Greenbaum CJ, Bundy B, Becker DJ, Gitelman SE, Goland R (2014). B-lymphocyte depletion with rituximab and β-cell function: two-year results. Diabetes Care.

[20] Herold KC, Gitelman S, Greenbaum C (2009). Immune tolerance network ITN007AI study group treatment of patients with new onset type 1 diabetes with a single course of anti-CD3 mAb Teplizumab preserves insulin production for up to 5 years. Clin Immunol.

[21] Keymeulen B, Walter M, Mathieu C (2010). Four-year metabolic outcome of a randomised controlled CD3-antibody trial in recent-onset type 1 diabetic patients depends on their age and baseline residual beta cell mass. Diabetologia..

[22] Orban T, Bundy B, Becker DJ (2014). Type 1 diabetes TrialNet Abatacept study group. Co-stimulation modulation with abatacept in patients with recent-onset type 1 diabetes: follow-up one year after cessation of treatment. Diabetes Care.

[23] Nakahara C, Kamoda T, Kinugasa H (2000). Simultaneous onset of nephrotic syndrome and insulin-dependent diabetes mellitus in a case with hypereosinophilia syndrome. Clin Nephrol.

[24] Agras PI, Kinik ST, Cengiz N, Baskin E (2006). Type 1 diabetes mellitus associated with nephrotic syndrome. J Pediatr Endocrinol Metab.

[25] Kari JA, El-Desoky SM, Mokhtar G, Jalalah SM. Simultaneous onset of steroid resistant nephrotic syndrome and IDDM in two young children. BMJ Case Rep. 2010:bcr0420102916. 10.1136/bcr.04.2010.2916. 10.1136/bcr.04.2010.2916 PMC3030072 22798086

[26] Peces R, Riera JR, Lopez Larrea C, Alvarez J (1987). Steroid-responsive relapsing nephrotic syndrome associated with early diabetic glomerulopathy in a child. Nephron..

[27] Kagiyama S, Tsuruta H, Tominaga M, Morishita K, Doi Y, Onoyma K (1999). Minimal-change nephrotic syndrome and acute renal failure in a patient with aged onset insulin-dependent diabetes mellitus and autoimmune thyroiditis. Am J Nephrol.

[28] Kawasaki E, Eguchi K (2004). Is type 1 diabetes in the Japanese population the same as among Caucasians?. Ann N Y Acad Sci.

[29] Kawabata Y, Ikegami H, Kawaguchi Y, Fujisawa T, Shintani M, Ono M (2002). Asian-specific HLA haplotypes reveal heterogeneity of the contribution of HLA-DR and -DQ haplotypes to susceptibility to type 1 diabetes. Diabetes..

[30] Jia X, Horinouchi T, Hitomi Y, Shono A, Khor S, Omae Y (2018). Strong association of the HLA-DR/DQ locus with childhood steroid-sensitive nephrotic syndrome in the Japanese population. J Am Soc Nephrol.

[31] Koyama A, Fujisaki M, Kobayashi M, Igarashi M, Narita M (1991). A glomerular permeability factor produced by human T cell hybridomas. Kidney Int.

[32] Pereira Wde F, Brito-Melo GE, Guimaraes FT, Carvalho TG (2014). The role of the immune system in idiopathic nephrotic syndrome. A review of clinical and experimental studies. Inflamm Res.

[33] Araya CE, Wasserfall CH, Brusko TM, Mu W, Segal MS, Johnson RJ, Garin EH (2006). A case of unfulfilled expectations. Cytokines in idiopathic minimal lesion nephrotic syndrome. Pediatr Nephrol.

[34] Kanai T, Shiraishi H, Yamagata T, Ito T, Odaka J, Saito T, Aoyagi J, Momoi MY (2010). Th2 cells predominate in idiopathic steroid-sensitive nephrotic syndrome. Clin Exp Nephrol.

[35] Azar ST, Tamim H, Beyhum HN, Habbal MZ, Almawi WY (1999). Type 1 (insulin-dependent) diabetes is a Th1- and Th2-mediated autoimmune disease. Clin Diagn Lab Immunol.

[36] Meadow SR, Sarsfield JK, Scott DG, Rajah SM (1981). Steroid-responsive nephrotic syndrome and allergy: immunological studies. Arch Dis Child.

[37] Riley WJ (1992). Autoimmune polyglandular syndromes. Horn Res.

[38] Almawi WY, Tamim H, Azar ST (1999). T helper type 1 and 2 cytokines mediate the onset and progression of type 1 (insulin-dependent) diabetes. J Clin Endocrinol Metab.

[39] Colucci M, Corpetti G, Emma F, Vivarelli M (2018). Immunology of idiopathic nephrotic syndrome. Pediatr Nephrol.

[40] Viardot A, Grey ST, Mackay F, Chisholm D (2007). Potential antiinflammatory role of insulin via the preferential polarization of effector T cells toward a T helper 2 phenotype. Endocrinology.

[41] Gladstone P, Nepom GT (1995). The prevention of IDDM. Injecting insulin into the cytokine network. Diabetes..

[42] Kretowski A, Myśliwiec J, Szelachowska M, Kinalski M, Kinalska I (1999). Insulin increases in vitro production of Th2 profile cytokines in peripheral blood cultures in subjects at high risk of diabetes type 1 and patients with newly diagnosed IDDM. Horm Metab Res.

