# Supplementary material for: Concurrent minimal change nephrotic syndrome and type 1 diabetes mellitus in an adult Japanese woman: a case report
Source: BMC Nephrol. 2020 Sep 23;21:410. doi: 10.1186/s12882-020-02071-6 (PMC7510261; doi:10.1186/s12882-020-02071-6)
Supplement: Supplementary file 1 — Additional file 1. [file 12882_2020_2071_MOESM1_ESM.pptx]

## Slide 1
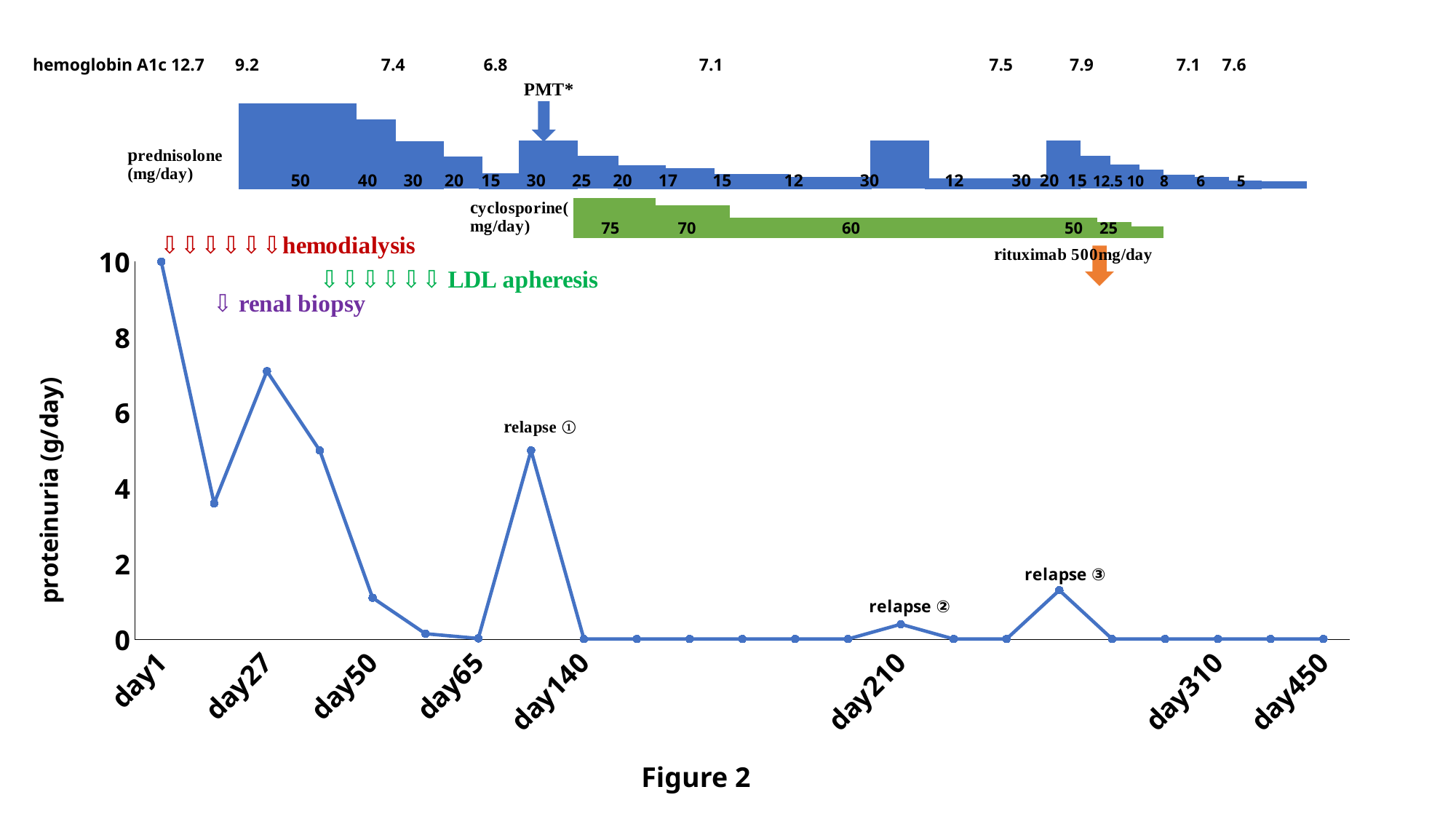

hemoglobin A1c 12.7 9.2 7.4 6.8 7.1 7.5 7.9 7.1 7.6
### Chart
| Category | proteinuria |
|---|---|
| day1 | 10.0 |
| day13 | 3.6 |
| day27 | 7.1 |
| day41 | 5.0 |
| day50 | 1.1 |
| day57 | 0.15 |
| day65 | 0.025 |
| day120 | 5.0 |
| day140 | 0.01 |
| | 0.01 |
| | 0.01 |
| day170 | 0.01 |
| | 0.01 |
| | 0.01 |
| day210 | 0.4 |
| | 0.01 |
| | 0.01 |
| day260 | 1.3 |
| | 0.01 |
| | 0.01 |
| day310 | 0.01 |
| | 0.01 |
| day450 | 0.01 |50 40 30 20 15 30 25 20 17 15 12 30 12 30 20 15 12.5 10 8 6 5
75 70 60 50 25
Figure 2
